# Mapping of Plasma Membrane Proteins Interacting With *Arabidopsis thaliana* Flotillin 2

**DOI:** 10.3389/fpls.2018.00991

**Published:** 2018-07-12

**Authors:** Petra Junková, Michal Daněk, Daniela Kocourková, Jitka Brouzdová, Kristýna Kroumanová, Enric Zelazny, Martin Janda, Radovan Hynek, Jan Martinec, Olga Valentová

**Affiliations:** ^1^Department of Biochemistry and Microbiology, University of Chemistry and Technology Prague, Prague, Czechia; ^2^Institute of Experimental Botany of the Czech Academy of Sciences, Prague, Czechia; ^3^Department of Experimental Plant Biology, Faculty of Science, Charles University, Prague, Czechia; ^4^Institut de Biologie Intégrative de la Cellule (I2BC), CNRS–CEA–Université Paris Sud, Université Paris-Saclay, Gif-sur-Yvette, France

**Keywords:** Arabidopsis flotillin 2, protein–protein interactions, immunopurification, mass spectrometry, split-ubiquitin yeast system, plant–pathogen interaction, water transport, intracellular trafficking

## Abstract

Arabidopsis flotillin 2 (At5g25260) belongs to the group of plant flotillins, which are not well characterized. In contrast, metazoan flotillins are well known as plasma membrane proteins associated with membrane microdomains that act as a signaling hub. The similarity of plant and metazoan flotillins, whose functions most likely consist of affecting other proteins via protein–protein interactions, determines the necessity of detecting their interacting partners in plants. Nevertheless, identifying the proteins that form complexes on the plasma membrane is a challenging task due to their low abundance and hydrophobic character. Here we present an approach for mapping *Arabidopsis thaliana* flotillin 2 plasma membrane interactors, based on the immunoaffinity purification of crosslinked and enriched plasma membrane proteins with mass spectrometry detection. Using this approach, 61 proteins were enriched in the *AtFlot-GFP* plasma membrane fraction, and 19 of them were proposed to be flotillin 2 interaction partners. Among our proposed partners of Flot2, proteins playing a role in the plant response to various biotic and abiotic stresses were detected. Additionally, the use of the split-ubiquitin yeast system helped us to confirm that plasma-membrane ATPase 1, early-responsive to dehydration stress protein 4, syntaxin-71, harpin-induced protein-like 3, hypersensitive-induced response protein 2 and two aquaporin isoforms interact with flotillin 2 directly. Based on the results of our study and the reported properties of Flot2 interactors, we propose that Flot2 complexes may be involved in plant–pathogen interactions, water transport and intracellular trafficking.

## Introduction

The SPFH (stomatin/prohibitin/flotillin/HflK/C) domain proteins superfamily consists of membrane proteins which exhibit 40–84% sequence homology (Li et al., 2000; Green and Young, 2008), but are divided into several groups with different functions and localizations (Browman et al., 2007). Flotillins form a group of SPFH domain-containing proteins characterized by their localization in the plasma membrane.

Flotillins were discovered in three independent studies as a human epidermal surface antigen (Schroeder et al., 1994), as proteins induced during optic nerve regeneration in the goldfish retinal ganglion (Schulte et al., 1995) and as proteins of membrane caveolae in a mouse fibroblast tissue culture (Bickel et al., 1997). To this day, the localization and function of metazoan flotillins has been intensively investigated. Metazoan flotillins are predominantly targeted to plasma membrane microdomains, where they are anchored by the SPFH domain (Morrow et al., 2002; Neumann-Giesen et al., 2004; Glebov et al., 2006; Solis et al., 2007; Langhorst et al., 2008). They were found to be involved in the endocytosis of glycophosphatidylinositol (GPI)-anchored proteins as well as caveolae-mediated endocytosis (Volonté et al., 1999; Baumann et al., 2000). Another function of flotillins likely consists of affecting other proteins via protein–protein interactions (Baumann et al., 2000), in which case various tyrosine kinases (Ullrich and Schlessinger, 1990; Neumann-Giesen et al., 2007; Amaddii et al., 2012) or proteins of the cytoskeleton (Baumann et al., 2000; Liu et al., 2005; Langhorst et al., 2008; Peremyslov et al., 2013) are prominent interactors with mammalian flotillins. The interaction with partner proteins as well as homo- and hetero-oligomerization of single metazoan flotillin isoforms is predominantly provided by the C-terminal domain of flotillins, where several coiled-coil stretches are present (Neumann-Giesen et al., 2004; Solis et al., 2007).

The coding regions of flotillin homologs were also identified in various plant genomes (Di et al., 2010). For example, the *A. thaliana* genome contains three homologs of flotillin, Flot1 (At5g25250), Flot2 (At5g25260), and Flot3 (At5g64870) (Gehl et al., 2014; Jarsch et al., 2014) and in this paper these three isoforms are designated Flot1/2/3 unless stated otherwise. Similarly to metazoan homologs, Arabidopsis flotillins are able to form heterooligomers via their C-terminal domain, which was reported for the direct interaction of Flot1 with Flot3 (Yu et al., 2017). However, the role of plant flotillins, as well as of most other proteins with a SPFH domain, has not been fully elucidated. Current findings about the localization and function of plant flotillins in the context of the known role of metazoan flotillins have been recently summarized by Danek et al. (2016). Similarities between the properties of plant and metazoan flotillins lead to the assumption that plant flotillins affect other proteins via protein–protein interactions, as with metazoans.

*Arabidopsis thaliana* flotillins differ in the localization of their transcription, because Flot1 and Flot2 are predominantly transcribed in leaves and shoots, while Flot3 is mostly transcribed in the flower parts and siliques (Danek et al., 2016). Nevertheless, the subcellular localization is similar for all known flotillins; they are most frequently localized to plasma membrane microdomains (Li et al., 2012; Hao et al., 2014; Jarsch et al., 2014; Ishikawa et al., 2015), which are enriched in sterols, sphingolipids, saturated phospholipids and GPI-anchored proteins, and play a significant role in membrane trafficking and cell signaling (Simons and Ikonen, 1997; Simons and Toomre, 2000; Borner et al., 2005; Jarsch et al., 2014; Cacas et al., 2016).

Although the anchoring of mammalian flotillins is supported by their palmitoylation as well as myristoylation (Morrow et al., 2002; Neumann-Giesen et al., 2004; Langhorst et al., 2008), no sites for palmitoylation or myristoylation were predicted in any of the three *A. thaliana* flotillins. This indicates that the anchoring to the membrane is provided by a different mechanism (Danek et al., 2016). This mechanism could be based on the specific interaction with sterols, since several putative CRAC/CARC motifs providing recognition and interaction with sterols were predicted in the sequence of plant flotillins (Roitbak et al., 2005; Danek et al., 2016). This hypothesis is supported by the finding that the Flot1 diffusion coefficient is decreased in plants treated with methyl-β-cyclodextrin, a sterol-depleting agent (Li et al., 2011, 2012; Hao et al., 2014). Moreover, it was also observed that the knocking-down of *Flot1* affected the internalization of sterol into membranes (Li et al., 2012).

Since proteins involved in vesicular trafficking and endocytosis (e.g., ESCRT proteins, exocyst and SNARE subunits or Rab-GTPase) were proposed to be Flot2 and Flot3 interactors by Associomics, a split-ubiquitin yeast system-based database of direct protein–protein interactions^1^ (Jones et al., 2014), this suggests that plant flotillins could play a similar role in membrane transport to mammalian ones. Additionally, plant flotillin microdomains have been shown to be involved in clathrin-independent endocytosis, inducible by various stimuli (Li et al., 2011; Hao et al., 2014; Wang et al., 2015; Yu et al., 2017). The role of flotillins in cell communication and signal transduction is also considered, because several types of kinases were found to co-localize with *Medicago truncatula* Flot4 (Haney et al., 2011) and interact with all three *AtFlot* isoforms (Associomics). The involvement of flotillins in plant–pathogen interactions was demonstrated, as Flot1 lateral mobility in the plasma membrane was altered upon treatment with bacterial elicitor flg22, and reduced or increased flg22-induced callose deposition was observed in plants with *Flot1* knocked-down or overexpressed, respectively (Yu et al., 2017). Arabidopsis amiRNA-Line with reduced *Flot1/Flot2* expression were smaller in size and exhibited structural changes in apical meristems (Li et al., 2012), which points to the involvement of flotillins in plant growth and development. Moreover, functional linkage and co-localization of plant flotillins and the cytoskeleton was observed (Li et al., 2012; Peremyslov et al., 2013).

Protein interactions with other cell components are crucial to maintaining the viability of the whole organism and determining its phenotypic manifestation. Predominantly, protein–protein interactions are nowadays intensively examined by various methods. Among them, immunoprecipitation (IP) or affinity purification (AP) coupled to mass spectrometry (MS) is the method of choice (ten Have et al., 2011; Dunham et al., 2012; Dedecker et al., 2015). However, the investigation of membrane proteins is challenging due to their hydrophobic character and low abundance. Nevertheless, there is a current effort to modify standard procedures in order to facilitate analyses of membrane protein interactions (Qi and Katagiri, 2009; Smaczniak et al., 2012; Van Leene et al., 2015).

The aim of this study was to perform a screening of Flot2 protein interactors in *A. thaliana* leaves. For this purpose we used IP with a GFP tag followed by the MS of *in vitro* cross-linked membrane proteins. After we confirmed the localization of Flot2 at the plasma membrane, we showed that the enrichment of the plasma membrane prior to the IP-MS is a crucial step for the detection of low–abundance plasma membrane interactors. The direct interaction of Flot2 with several proteins involved in the plant response to biotic as well as abiotic stress was confirmed by an independent method, e.g., by a split-ubiquitin yeast system (SUS) suitable for the analysis of membrane proteins.

## Materials and Methods

### Plant Material

Transgenic *A. thaliana* lines *AtFlot2-GFP* with the p35S::AtFlot2:GFP construct that stably produces the Flot2-GFP protein were prepared as follows: The coding sequence was amplified from cDNA prepared from Col-0 using specific primers 1 and 2 (see Supplementary Table S1) and in-frame introduced in between the EcoRI and BamHI sites of a modified pGreen0029 vector containing the CAMV 35S promoter and 3′-terminal GFP coding sequence by restriction/ligation. Stable transformants were obtained by the *Agrobacterium tumefaciens* floral dip method and selected on kanamycin plates. T3 generation plants were used for microscopy and membrane fractions preparation.

Seeds of the *A. thaliana* wild type (WT, ecotype Col-0) and *AtFlot2-GFP* plants were stratified for 3 days at 4°C, placed on Jiffy 7 peat pellets and cultivated in a growth chamber at 22°C, with a 10-h day (100–130 μmol m^-2^ s^-1^) and 14-h night cycle at 70% relative humidity for 1 week. One-week-old plantlets were individually replanted to Jiffy 7 peat pellets and placed in a cultivation room with a 16-h day (100–130 μmol m^-2^ s^-1^) and 8-h night cycle and 40–50% relative humidity. During the cultivation, plants were watered with distilled water. Whole rosettes of 4-week-old plants were frozen in liquid nitrogen to be used as the material for MS analyses.

### Confocal Microscopy

For microscopic observations, seeds of *AtFlot2-GFP* plants were surface sterilized and sown onto Murashige-Skoog basal salt (Duchefa) 1% agar plates supplemented with 1% sucrose. The seedlings were grown in a vertical position under 100 μmol m^-2^ s^-1^ in a 16/8 h and 22/20°C (light/dark) cycle. Five-day-old seedlings were observed using a Zeiss 880 laser scanning confocal microscope. Plasmolysis was induced by treatment with 0.8 M mannitol in Murashige-Skoog solution for 30 min. Subsequently, the seedlings were incubated in propidium iodide solution (20 μg/ml in Murashige-Skoog + 0.8 M mannitol) to counterstain the cell walls. GFP fluorescence was collected in the 500–550 nm range using 488 nm laser excitation and a 40× water immersion objective (NA = 1.2).

### Preparation of Microsomal and Plasma Membrane Fractions

Membrane fractions were prepared from 30 g of leaves from 4-week-old *A. thaliana* WT and *AtFlot2-GFP* plants. Leaves were ground with a pestle and mortar in liquid nitrogen and further homogenized by sonication for 3 × 35 s (25 W) in 90 ml of extraction buffer (50 mM HEPES pH 7.5, 400 mM sucrose, 85 mM KCl, 100 mM MgCl_2_.6H_2_O, 0.02 mM ascorbic acid) containing cOmplete^TM^ EDTA-free Protease Inhibitor Cocktail according to the manufacturer’s instructions (Sigma Aldrich). The homogenate was centrifuged at 5000 ×*g* for 20 min at 4°C. The supernatant was filtered through Miracloth (Millipore) and centrifuged at 200,000 ×*g* for 1 h at 4°C. The pellet (microsomal membrane fraction) was resuspended in resuspension buffer (20 mM HEPES pH 7.5, 330 mM sucrose, 1 mM EDTA) to a total volume of 6 ml, further homogenized in a Potter-Elvehjem homogenizer and cross-linked by the addition of dithiobis (succinimidyl propionate) (DSP, Thermo Scientific) to a final concentration of 5 mM. The suspension was incubated for 30 min at 4°C with shaking. To quench the reaction, 1 M Tris (pH 7.5) was added to a final concentration of 50 mM, and the suspension was shaken again for 30 min at 4°C. The microsomal fraction with cross-linked proteins was pelleted by centrifugation at 200,000 ×*g* for 1 h at 4°C.

To release the protein complexes from the microsomal fraction, the pellet was resuspended in 6 ml of resuspension buffer and homogenized with a Potter-Elvehjem homogenizer, and then 10% (w/v) sodium deoxycholate was added to a final concentration of 0.5% (w/v). The suspension was incubated for 30 min at 4°C. Solubilized proteins were collected in the supernatant obtained by centrifugation at 200,000 ×*g* for 30 min at 4°C, and the pellet was resuspended in the same way as before. To further enrich the plasma membrane, the pellet of the cross-linked microsomal fraction was resuspended in 5 mM K/Na-phosphate buffer (pH 7.8) and homogenized with a Potter-Elvehjem homogenizer.

The plasma membrane fraction was prepared from the cross-linked membrane fraction with a PEG/dextran two-phase system (Schindler and Nothwang, 2006; Pleskot et al., 2010). After gentle mixing, the separation was carried out overnight at 4°C, and the tubes were centrifuged at 1500 ×*g* for 5 min at 4°C. The upper phase containing the plasma membrane was transferred to the new tubes, mixed with a blank lower phase and centrifuged again. The final upper phase was collected, diluted with three volumes of 5 mM K/Na-phosphate buffer (pH 7.8) and centrifuged again at 200,000 ×*g* for 1 h at 4°C. The pellet was resuspended in 600 μl of resuspension buffer, 10% (w/v) sodium deoxycholate was added to a final concentration of 0.5% (w/v), and the suspension was incubated for 30 min at 4°C. The protein content in all isolated fractions was determined by Popov’s method (Popov et al., 1975) using bovine serum albumin as the standard. Flow chart of the procedure is depicted on **Figure 2A**.

### Western Blotting and Immunodetection

The content of Flot2-GFP in the respective fractions was investigated by western blotting and immunodetection. Proteins were separated in 10% polyacrylamide SDS-gels at 180 V and electroblotted onto nitrocellulose membranes (BioTrace^TM^ NT Nitrocellulose Transfer Membrane, Pall Corporation) at 50 V. Membranes were rinsed in PBS and blocked in 5% (w/v) non-fat milk powder in PBS with 0.075% (w/v) Tween-20 (PBST-75) overnight. Blocked membranes were washed three times in PBST-75 and incubated with primary antibodies diluted in 5% (w/v) non-fat milk powder in PBST-75 for 1 h. Anti-GFP rabbit polyclonal serum (Thermo Scientific) 1:5000 was used as the primary antibody. Membranes were washed three times in PBST-75 and incubated for 1 h with the secondary antibody, GAR/IgG(H + L)/PO (Nordic-MUbio) 1:5000 diluted in 5% (w/v) non-fat milk powder in PBST-75. Signals were visualized with an AEC staining kit (Sigma Aldrich) or Clarity^TM^ Western ECL Substrate (Bio-rad).

### Immunoprecipitation of Microsomal and Plasma Membrane Fractions

The IP procedure was performed with Dynabeads^®^ Protein A microbeads (Thermo Scientific) with bound anti-GFP mouse monoclonal antibody, isotype IgG_2a_ (Thermo Scientific). Fifty microliter of pre-washed beads were mixed with 2 μg of antibodies dissolved in PBS containing 0.05% Tween-20 (PBST-5) and incubated for 1 h in the vertical rotator. Beads with bound antibodies were washed three times with 200 μl of PBST-5 and incubated in 5 mM bis(sulfosuccinimidyl)suberate (BS3, Thermo Scientific) for 30 min to cross-link the bound antibodies to protein A. The reaction was quenched by washing the beads with 200 μl of 1 M Tris/HCl (pH 7.4) three times. The beads were then equilibrated three times with 200 μl of resuspension buffer with 0.5% (w/v) sodium deoxycholate and incubated with 200 μl of the respective membrane fraction for 2 h in a vertical rotator. Protein complexes bound to the beads were washed three times with RIPA buffer (50 mM Tris/HCl pH 7.4, 1 mM EDTA, 50 mM NaCl, 0.5% (w/v) sodium deoxycholate, 1% (w/v) NP-40) and eluted by incubation of the beads with 20 μl of Laemmli buffer 2× for 10 min at 95°C.

### Tryptic Digestion of Proteins

Proteins eluted from the microbeads were separated to a distance of 1.5 cm in 10% poly-acrylamide SDS-gels at 180 V. Gels were stained with Imperial^TM^ Protein Stain (Thermo Scientific) and whole line of each elute of was collected. Each lane was further sliced into smaller gel pieces and combined into an Eppendorf tube, washed with water, destained with 0.1 M NH_4_HCO_3_/acetonitrile 1:1 (v/v) and dried with acetonitrile. To reduce and alkylate the disulphide bonds, the gel pieces were first incubated with a 10 mM solution of dithiothreitol in 0.1 M NH_4_HCO_3_ for 45 min at 56°C, and then in a 55 mM solution of iodoacetamide in 0.1 M NH_4_HCO_3_ for 30 min at room temperature. Iodoacetamide solution was discarded and the gel pieces were washed with 0.1 M NH_4_HCO_3_/acetonitrile 1:1 (v/v) for 10 min and dried with acetonitrile. MS-Grade Trypsin solution at a concentration of 12.5 μg ml^-1^ dissolved in cold 50 mM NH_4_HCO_3_ was added to the gel pieces in a volume equal to the volume of the pieces, and the mixture was incubated on ice for 30 min. The excess trypsin solution was then discarded; the pieces were covered with 50 mM NH_4_HCO_3_ and incubated overnight at 37°C. The peptides were extracted from the gel by two consecutive sonications in 35 and 70% solutions of acetonitrile in 0.1% trifluoroacetic acid. Both aliquots were combined and the resulting peptide solution was lyophilized. The lyophilizate was then resuspended in 0.1% trifluoroacetic acid, desalted with ZipTip pipette tips according to the manufacturer’s instructions (Millipore) and purified samples were dried in air.

### LC-MS/MS Analysis

The mass spectrometric analysis was performed with a UHPLC Dionex Ultimate3000 RSLC nano (Dionex) coupled with an ESI-Q-TOF Maxis Impact (Bruker Daltonics) mass spectrometer. Dried samples were dissolved in a mixture of water:acetonitrile:formic acid (97:3:0.1%) and loaded into the trap column, an Acclaim PepMap 100 C18 (100 μm × 2 cm, particle size 5 μm, Dionex), with a mobile-phase flow rate of 5 μLmin^-1^ of A (0.1% formic acid in water) for 5 min. The peptides were then separated in the analytical column, an Acclaim PepMap RSLC C18 (75 μm × 150 mm, particle size 2 μm, Dionex), and eluted with mobile-phase B (0.1% formic acid in acetonitrile) using the following gradient: 0 min 3% B, 5 min 3% B, 95 min 35% B, 97 min 90% B, 110 min 90% B, 112 min 3% B, and 120 min 3% B. The flow rate during the gradient separation was set to 0.3 μLmin^-1^. Peptides were eluted directly to the ESI source-captive spray (Bruker Daltonics). Measurements were performed in DDA mode with precursor-ion selection in the range of 400–1400 Da; up to 10 precursor ions were selected for fragmentation from each MS spectrum.

Peak lists were extracted from the raw data with the software Data Analysis 4.1 (Bruker Daltonics). Proteins were identified in the software Proteinscape 3.1 (Bruker Daltonics) using in-house Mascot server 2.4.1 (Matrix Science) with the *A. thaliana* protein database downloaded from^2^ (October 2016). The parameters for the database search were set as follows: carbamidomethyl (C) as fixed modification, oxidation (M) and CAMthiopropanoyl (K, N-terminus) as variable modifications, tolerance 10 ppm in MS mode and 0.05 Da in MS/MS mode, enzyme trypsin one miscleavage. In MS intensity-based semiquantitative analysis the relative intensities of unique peptide signals were averaged to express individual protein abundance. Only proteins identified by two or more peptides were taken into account and the intensities of precursor ions with the best mascot score were used. Finally, the relative quantification index (RQI) representing the ratio of the resulting protein abundance between the *AtFlot2-GFP* plant sample and WT sample was calculated for each protein, and the proteins with RQI higher than three were considered to be enriched.

### Split-Ubiquitin System

The Flot2 coding sequence was amplified from cDNA prepared from Col-0 using specific primers 3 and 4 (see Supplementary Table S1) and introduced in between the SalI and NotI restriction sites of the pENTR3c Dual Selection vector (Thermo Fisher) in a manner that allowed C-terminal protein fusion by restriction/ligation. LR recombination with the pMetYC-DEST vector encoding the C-terminal split-ubiquitin moiety as well as the LEU2 gene was then performed using Gateway^TM^ LR Clonase^TM^ II Enzyme mix (Thermo Fisher) to obtain the final vector for yeast transformation.

Putative interactor coding sequences were amplified from cDNA prepared from Col-0 using specific primers 5–26 (see Supplementary Table S1) and introduced in between the KpnI and NotI (AtPIP2-6 and AtSYP71) or SalI and NotI (the rest of the sequences) restriction sites of the pENTR3c Dual Selection vector (Thermog Fisher) in a manner that allowed N-terminal protein fusion by restriction/ligation. LR recombination with a pNX35-DEST vector encoding the N-terminal split-ubiquitin moiety as well as the TRP1 gene was then performed using Gateway^TM^ LR Clonase^TM^ II Enzyme mix (Thermo Fisher) to obtain the final vector for yeast transformation.

The THY.AP4 yeast strain was cotransformed (Hachez et al., 2014) with Flot2-pMetYC and X-pNX35 (X = investigated putative interactor of Flot2) vectors by the lithium acetate/single-stranded carrier DNA/PEG method (Grefen et al., 2009; Grefen, 2014) and plated on YNB + CSM (both MP Biomedicals) medium lacking Leu and Trp supplemented with 2% glucose and 50 μM Met. After a 2-day recovery at 30°C, freshly grown colonies were resuspended in milliQ water and diluted to obtain suspensions of optical densities (OD_600_) equal to 1.0, 0.1, and 0.01. Drops of 10 μl were placed on plates with YNB + CSM selective medium lacking Leu, Trp, Ade, and His, supplemented with 2% glucose and 50, 250, and 500 μM Met. Yeast growth was visually assessed after incubation for 2 days at 30°C.

The non-recombined pNX35 vector encoding for NubG, which was unable to reassemble with Cub co-transformed with Flot2-pMetYC, was used as the negative control, whereas the pNubWT-Xgate vector encoding for the wild-type Nub moiety spontaneously reassembling with Cub cotransformed with Flot2-pMetYC was used as the positive control in the SUS growth assay.

The pMetYC-DEST, pNX35-DEST, and pNubWT-Xgate vectors as well as the THY.AP4 yeast strain were kindly provided by Christopher Grefen, University of Tubingen, Germany. AtPIP2-7-pNX32 was kindly provided by François Chaumont and Timothée Laloux, Université catholique de Louvain, Belgium.

## Results

### Plasma Membrane Localization of Flot2-GFP

**FIGURE 1:**
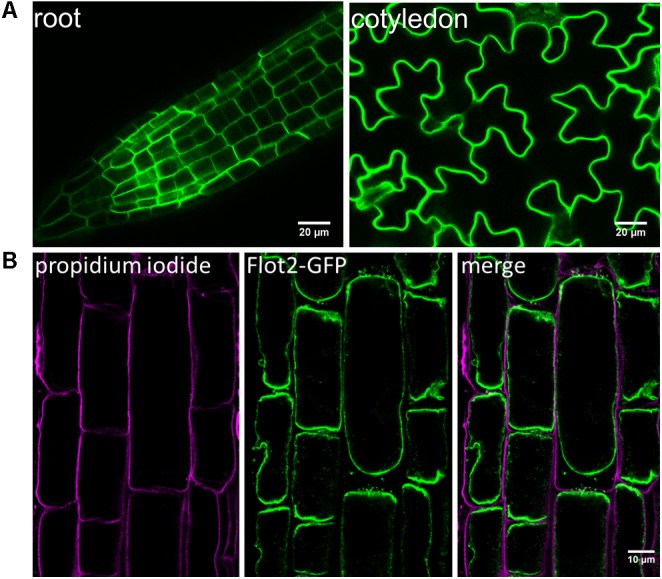
Flot2-GFP localization at plasma membrane. **(A)** Confocal microscopy images showing Flot2-GFP localization at plasma membrane in epidermal cells of roots and cotyledons of Arabidopsis Flot2-GFP plants; **(B)** Confirmation of Flot2-GFP localization at plasma membrane in plasmolyzed root epidermal cells. The Flot2-GFP signal is detected at the plasma membrane of contracted protoplasts. Seedlings were treated with 0.8 M mannitol and subsequently stained with propidium iodide to mark cell walls.

Since the only experimental evidence of the subcellular localization of Flot2 at the plasma membrane was found when YFP-fused *A. thaliana* Flot2 was transiently expressed in *Nicotiana benthamiana* leaf epidermal cells (Jarsch et al., 2014), we investigated the localization of Flot2-GFP directly in the epidermal cells of *A. thaliana* roots and cotyledons (**Figure 1A**). We observed that Flot2-GFP is predominantly localized at the plasma membrane in both of these diverse *A. thaliana* tissues. Flot2-GFP localization at plasma membrane was confirmed by subjecting root cells to the plasmolysis induced by mannitol; no Flot2-GFP signal was detected at the cell wall (**Figure 1B**). Therefore, not only the microsomal fraction, but also the enriched plasma membrane fraction was prepared to perform the IP-MS experiment. The lines overexpressing Flot2-GFP did not exhibit any apparent growth differences from Col-0 plants.

### Enrichment of Flot2-GFP in Plasma Membrane Fractions

**FIGURE 2:**
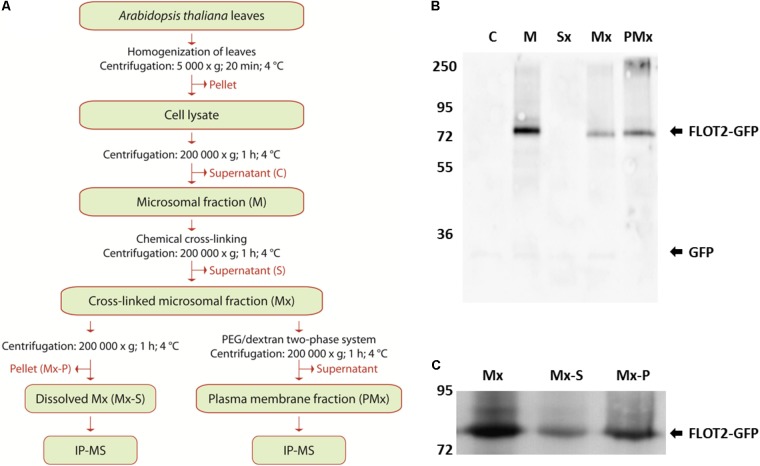
Preparation and characterization of Flot2-GFP membrane fractions. **(A)** Flow chart of the preparation and analysis of membrane fractions; **(B)** Immunoblot analysis of Flot2-GFP content in cytosolic fraction (C), microsomal fraction (M), supernatant obtained after pelleting of cross-linked microsomal fraction (Sx), cross-linked microsomal fraction (Mx), plasma membrane fraction isolated from cross-linked microsomal fraction (PMx), 0.5 μg of total proteins were loaded into all lines; **(C)** Immunoblot analysis of Flot2-GFP content in whole cross-linked microsomal fraction (Mx), fraction dissolved by 0.5% (w/v) sodium deoxycholate (Mx-S) and fraction undissolved by 0.5% (w/v) sodium deoxycholate (Mx-P), 10 μg of total proteins were loaded into all lines.

The microsomal fraction was isolated according to Qi and Katagiri (2009). A DSP cross-linker was used to fix the interacting proteins in the microsomal fraction before the dissolution of membranes with 0.5% (w/v) sodium deoxycholate. Due to the plasma membrane localization of Flot2, we also enriched the plasma membrane fraction with an extract from the cross-linked microsomal fraction, because the direct determination of its plasma membrane interactors could better contribute to the characterization of Flot2’s function. A flow chart of the procedure is depicted in **Figure 2A**.

During the isolation process, the presence of Flot2-GFP in each obtained fraction was detected by immunoblot analysis using the antibodies against GFP. Significant loss of the Flot2-GFP content caused by additional ultracentrifugation was observed between the native (line M) and cross-linked (line Mx) microsomal fraction (**Figure 2B**). We also analyzed the content of Flot2-GFP in fractions obtained after the dissolution of the cross-linked microsomal fraction by sodium deoxycholate; a higher amount of Flot2-GFP remained in the undissolved fraction (**Figure 2C**). Nevertheless, the total protein as well as Flot2-GFP content in the dissolved microsomal fraction was sufficient to perform the IP-MS analysis.

During the isolation of the plasma membrane fraction, a significantly lower yield of total proteins was obtained compared to the yield of proteins in the microsomal fraction. Finally, only approximately 2 μg of total proteins per 1 g of initial material were obtained in the enriched plasma membrane fraction. Nevertheless, the signal of Flot2-GFP in the enriched plasma membrane fraction (PMx line) was more intense than its signal in the cross-linked microsomal fractions (Mx line), which indicates that Flot2-GFP was successfully enriched in the plasma membrane fraction (**Figure 2B**).

### Identification of Proteins Interacting With Flot2-GFP

Immunoprecipitation-MS of the microsomal as well as plasma membrane fraction was performed in nine repetitions. Only proteins which were detected at least three times in the immunoprecipitated *AtFlot2-GFP* membrane fractions and were not detected in the WT membrane fractions (control) were considered to be potential interactors of Flot2-GFP (**Table 1**). However, three additional proteins which were also detected in the control samples were included in the list of potential interactors, since they were significantly enriched in the *AtFlot2-GFP* sample according to MS intensity-based semiquantitative analysis. It can be seen in **Table 1** that the IP-MS of the microsomal fraction provided a substantially lower number of potential interactors than the plasma membrane fraction. In total, 16 proteins were detected in this fraction, and only three of those were proposed to be Flot2 interaction partners. The majority of the detected proteins were actually only detected in one or two repetitions (Supplementary Table S2a). On the other hand, 61 proteins were enriched in the *AtFlot-GFP* plasma membrane fraction (Supplementary Table S2b). Of those, 19 proteins were proposed to be Flot2 interaction partners (**Table 1**).

**Table 1:** Potential interactors of Arabidopsis flotillin 2.

F	Protein	Gene name	Locus	Counts		RQI
				*AtFlot2-GFP*	WT	
M	Flotillin 2	FLOT2	At5g25260	9	–	–
	Ubiquitin-60S ribosomal protein L40-1	RPL40A	At2g36170	5	–	–
	Aquaporin PIP2-1	PIP2-1	At3g53420	5	–	–
	Glyceraldehyde-3-phosphate dehydrogenase	GAPA1	At3g26650	3	–	–

PM	Flotillin 2	FLOT2	At5g25260	9	–	–
	Photosystem I reaction center subunit II-2, chloroplastic	PSAD2	At1g03130	5	–	–
	ATPase 1, plasma membrane-type	AHA1	At2g18960	3	–	–
	Early-responsive to dehydration stress protein	ERD4	At1g30360	3	–	–
	ABC transporter G family member 36	ABCG36	At1g59870	3	–	–
	Ubiquitin-60S ribosomal protein L40-1	RPL40A	At2g36170	3	–	–
	Aquaporin PIP1-2	PIP1-2	At2g45960	3	–	–
	Syntaxin-71	SYP71	At3g09740	3	–	–
	Aquaporin PIP2-2	PIP2-2	At2g37170	3	–	–
	Harpin-induced protein-like	NHL3	At5g06320	3	–	–
	Hypersensitive-induced response protein 2^∗^	HIR2	At3g01290	3	–	–
	Pyrophosphate-energized vacuolar membrane proton pump 1	AVP1	At1g15690	3	–	–
	Tubulin beta-5 chain	TUBB5	At1g20010	3	–	–
	5-methyltetrahydropteroyltri-glutamate-homocysteine methyltransferase 1	MS1	At5g17920	3	–	–
	Probable aquaporin PIP2-6	PIP2-6	At2g39010	3	–	–
	Probable inactive receptor kinase	–	At5g16590	3	–	–
	Aquaporin PIP2-1	PIP2-1	At3g53420	9	5	4.4
	Aquaporin PIP2-7	PIP2-7	At4g35100	8	3	3.5
	Carbonic anhydrase 2, chloroplastic	BCA2	At5g14740	7	1	3.2

*F, fraction; M, microsomal fraction; PM, plasma membrane fraction; Counts, number of IP repetitions (out of nine), in which the protein was detected; RQI, relative intensity index; ^∗^ At3g01290 is designated as HIR3 in UniProt database (www.uniprot.org)*.

**FIGURE 3:**
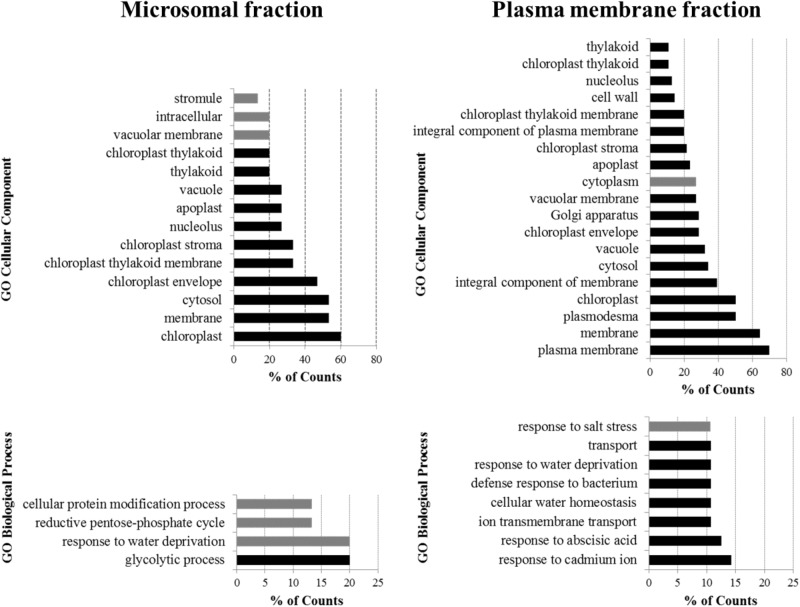
Cluster analysis of GO annotation terms of proteins enriched by IP of microsomal and plasma membrane fraction. The DAVID Bioinformatics Resources functional annotation clustering algorithm was used to cluster the most significant terms in the GO Cellular Component and GO Biological Process annotations. The terms are ordered from least frequent (top) to most frequent (bottom). Black bars indicate terms enriched with *p* < 0.01, gray bars indicate terms enriched with *p* ≥ 0.01.

To obtain greater insight into the proteins enriched by IP in both analyzed fractions (see Supplementary Table S2), a cluster analysis of GO annotation terms with respect to their localization and biological significance was performed using the DAVID Bioinformatics Resources annotation tool^3^ (Huang et al., 2009; **Figure 3**). Through the analysis of GO Cellular Component terms, it was found that the terms connected with the plasma membrane localization were only enriched when the plasma membrane fraction was used for the purification. This result shows the crucial importance of the appropriate fractioning of membrane proteins prior to their analysis. One of the most frequently occurring annotations of proteins purified from the plasma membrane fraction was localization in chloroplasts. When we mapped the proteins clustered within the chloroplast annotation, we found that half of them are simultaneously annotated to be localized in both, the plasma membrane and chloroplasts. Thus it is clear that the results of GO annotation cluster analysis can be influenced by the multiple annotations that exist for the proteins, and should therefore be carefully inspected.

According to the clustering of the GO Biological Process terms of proteins purified from the plasma membrane fraction, the potential interactors of Flot2 are predominantly involved in the plant response to various stress factors, such as the presence of cadmium ions, abscisic acid, bacteria, salt stress or water deprivation. Additionally, the response to water deprivation is the only process common to the proteins purified from both fractions.

### Verification of Flot2 Interactions by Split-Ubiquitin Yeast System

After having determined the potential Flot2 interactors, we applied the yeast SUS to test whether some of the revealed proteins interact directly with Flot2. In the system used, Flot2 was C-terminally fused with the C-terminal moiety of ubiquitin (Cub) and hybrid transcription factor PLV (ProteinA-LexA-VP16), which enables yeast growth on the selection medium. The methionine-repressible vector allows expression tuning in order to avoid false positive results (Grefen et al., 2007). Selected potential interactors of Flot2 were N-terminally fused to the N-terminal moiety of mutated ubiquitin (NubG), which is prevented from spontaneously reassembling with Cub.

**FIGURE 4:**
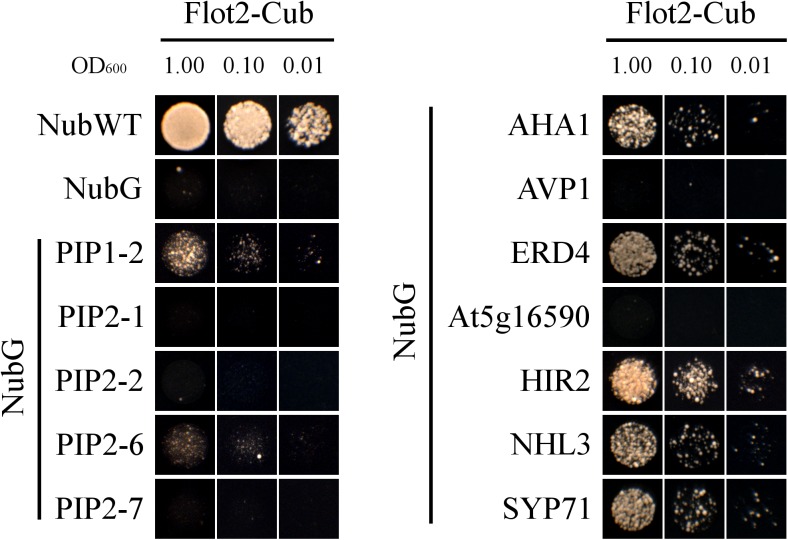
SUS test. Yeast strain THY.AP4 co-expressing Flot2-Cub-PLV and Nub fused with investigated possible interactors were plated in decimal dilution (OD_600_ = 1.00 or 0.10 or 0.01) onto selective media (without Ade, His, Leu, Trp) which was supplemented with 50, 250, or 500 μM Met in order to obtain a different expression level of the bait. Yeast growth rate was gradually repressed with an increasing concentration of Met. Only plates with 500 μM Met are presented. Plates were incubated for 48 h at 28°C. NubWT and NubG as prey were used as positive and negative controls, respectively. Similar growth performance was observed for each interaction with at least three biological replicates.

Twelve possible interactors from **Table 1** were investigated by SUS, of which seven gave positive results (**Figure 4**). Among the five plasma membrane aquaporins, yeast growth was only observed for PIP1-2 and PIP2-6. Nevertheless, the yeast growth was relatively weak for both PIPs. This suggests a weak or very transient interaction between PIPs and Flot2. The strong yeast growth apparent for plasma membrane ATPase 1 (AHA1), early-responsive to dehydration (ERD) stress protein (ERD4), hypersensitive-induced response protein 2 (HIR2), harpin-induced protein-like (NHL3) and syntaxin-71 (SYP71) demonstrate physical interaction with Flot2. The expression of aquaporins PIP2-1, PIP2-2, and PIP2-7, pyrophosphate-energized vacuolar membrane proton pump 1 (AVP1) and probable inactive receptor kinase (At5g16590) in co-transformed yeasts was confirmed (see Supplementary Figure 1) to rule out the possibility that the lack of yeast growth observed in these cases was caused by a lack of Nub-fused prey proteins. The positive expression of all five putative interactors implies that none of these proteins would directly interact with Flot2.

## Discussion

### Flot2-GFP Cellular Localization

Although metazoan flotillins were found to be most frequently localized to plasma membrane microdomains or endosomes (Baumann et al., 2000; Dermine et al., 2001; Glebov et al., 2006; Neumann-Giesen et al., 2007; Langhorst et al., 2008), they were also rarely detected in the mitochondria or nucleus of human cells (Santamaria et al., 2005; Ogura et al., 2014). In plants, a similar localization was observed in several studies, where the *A. thaliana* Flot1, *Picea meyeri* Flot1, and *Oryza sativa* Flot1 were enriched in the plasma membrane DRM fraction (Triton X-100-insoluble plasma membrane fraction) prepared from *A. thaliana* calli, spruce pollen tubes, and rice cells (Borner et al., 2005; Liu P. et al., 2009; Ishikawa et al., 2015). While Flot1 localization to the plasma membrane was confirmed by the confocal microscopy of *A. thaliana* stably transformed with *GFP-Flot1* (Li et al., 2012; Hao et al., 2014), the localization of Flot2 to the plasma membrane was only observed when YFP-fused Flot2 was transiently expressed in *N. benthamiana* leaf epidermal cells (Jarsch et al., 2014). Therefore, we investigated the localization of Flot2 directly in the epidermal cells of the roots and cotyledons of *A. thaliana* stably transformed with *Flot2-GFP*, and thus we confirmed its predominant localization to the plasma membrane (**Figure 1**).

### Enrichment of Flot2-GFP Containing Complexes

Due to the many difficulties associated with the IP/AP-MS of membrane protein complexes, new approaches are being investigated (Qi and Katagiri, 2009; Smaczniak et al., 2012; Huang and Kim, 2013; Dorr et al., 2016). Because of the low abundance of membrane proteins, there is a need to use harsh conditions in order to ensure their efficient solubilization and release from the membrane. To maintain protein interactions under these conditions, chemical cross-linkers providing a covalent binding of proteins in complexes could be used. With plant cell suspension cultures or seedlings, *in vivo* cross-linking can be performed using membrane-permeable cross-linkers such as DSP (Jafferali et al., 2014; Kaake et al., 2014). Nevertheless, *in vitro* cross-linking has also been successfully applied, where full-grown plants were used as input material (Qi and Katagiri, 2009; Qi et al., 2011). The chemical cross-linking of protein complexes also enables more stringent conditions to be used during IP/AP to eliminate the non-specific background.

Whole plants or their specific tissues, organs or cell compartments need to be analyzed when the plant development pathways or pathways specific for tissue, cell type or cell compartment are being studied (Ephritikhine et al., 2004; Tan et al., 2008). Nevertheless, the presence of abundant soluble proteins during the purification step can greatly contribute to an increase in false positives and, additionally, could be also problematic in MS-based approaches, where soluble proteins are favored over hydrophobic and low-abundant membrane proteins (Gilmore and Washburn, 2010). Therefore, the isolation of the whole membrane fraction or individual membrane compartments should be performed prior to purification (Speers and Wu, 2007; Qi and Katagiri, 2009; Savas et al., 2011).

In our study, we used both the *in vitro* cross-linking of membrane proteins in close proximity to each other to maintain the interactions in complexes during sample preparation, and the enrichment of Flot2-GFP in microsomes and the plasma membrane fraction prior to IP-MS. Compared to the results obtained by the IP-MS of the microsomal fraction, we were able to capture a substantially higher number of interactors localized to the plasma membrane by IP-MS of the enriched plasma membrane (**Figure 3**). Despite the large amount of initial plant material entering the analysis, due to the low abundance of plasma membrane within plant membranes, together with the substantial loss of plant material during the isolation of the plasma membrane from whole plants, we believe that this direct approach is indispensable for a better description of plasma membrane complexes.

### Determination of Flot2 Interactors

We used IP-MS to suggest potential interacting partners of Flot2. IP/AP-MS has become widely used nowadays, mainly thanks to the development of MS instrumentation that enables more efficient data acquisition. On the other hand, unfiltered IP/AP-MS data sets could give a large number of false positive interactions. To deal with this, a high number of repetitions and high number of controls should be analyzed (Pardo and Choudhary, 2012), different tags (Ho et al., 2002) as well as tag combinations (Van Leene et al., 2015) can be used, or some computational or informatics strategies can be applied for the evaluation of specific protein interactors (Collins and Choudhary, 2008; Choi H. et al., 2011; Nesvizhskii, 2012).

To identify specific interactors from the obtained IP-MS data set, some independent techniques such as Förster resonance energy transfer (FRET) or yeast two-hybrid assay can be used. In our study we suggested potential interactors by IP-MS and the specific interactors of Flot2 were then determined by SUS, a variant of yeast two-hybrid assay suitable for detecting a direct interaction between membrane-localized proteins.

Although SUS is far less used than the classical yeast two-hybrid test or bimolecular fluorescence complementation, it has been applied in more than 200 publications in major plant science journals to date (reviewed in Xing et al., 2016). Since SUS is a protein fragment complementation-based assay, there is a possibility of false positive (in comparison with e.g., FRET) as well as false negative results. To assess the possibility of false negative results (i.e., PIP2-1, PIP2-2, PIP2-7, AVP1, and At5g16590 in our study), it is necessary to keep in mind that the proper localization of both split ubiquitin moieties (to enable their reassembling at the cytoplasmatic side of the membrane) is a crucial prerequisite for the successful application of SUS. Therefore, the position of the N- or C-terminus of the investigated proteins (inside versus outside the cytoplasm) has to be considered when deciding, which protein terminus should be tagged with the split-ubiquitin moiety. In our study, all selected putative Flot2 interactors were fused at their N-terminus with NubG. In PIPs, both N- and C-terminal stretches are localized on the cytoplasmic sides of biomembranes (Murata et al., 2000; Luang and Hrmova, 2017), so tagging with NubG at each end is possible.

Nevertheless, proposing a suitable position for NubG fusion is tricky with AVP1 and At5g16590. AVP1 membrane topology prediction in tonoplasts suggests that both ends are localized inside vacuolar lumen (Pizzio et al., 2017). Hence, neither N- nor C-terminal fusion to NubG would be relevant for the interaction with cytoplasm-facing Cub. The structure of the At5g16590 protein is not published, and membrane-protein topology predictors do not provide unambiguous results (e.g., TMpred predicts the N-terminus in the cytoplasm while TMMOD predicts it on the extracellular side). On the other hand, At5g16590 belongs to a leucine-rich repeat kinase family, the majority of which have their N-terminal domains on the extracellular side of the plasma membrane (Diévart and Clark, 2003). However, several interactors were found for both NubG- and Cub-fused AVP1 and At5g16590 protein in Associomics. Thus, additional SUS assays with AVP1 and At5g16590 fused to NubG at their C-terminus will be necessary to further verify the interaction with Flot2 and potentially rule out the results found in this study as a false negative.

### Flot2 Interactors Are Found in Specific Plasma Membrane Subfractions

In our study, Flot2-GFP was found to be enriched in the deoxycholate-insoluble part of the microsomal fraction and the enriched plasma membrane fraction (**Figure 2**). Correspondingly most of the putative interactors found in our screen were already identified in membrane fractions resistant to mild detergents. AHA1, SYP71, NHL3, ERD4, PIP1-2, PIP2-7, and HIR2 as well as other HIR homologs HIR1 and HIR4 were enriched in the plasma membrane DRM fraction obtained from Arabidopsis plants, calli or suspension cells (Shahollari et al., 2004; Borner et al., 2005; Keinath et al., 2010). Moreover, homologs of SYP71, AHA1, and several PIPs were found in the plasma membrane DRM fraction from tobacco leaves (Mongrand et al., 2004). Although Flot2 was not identified in any of those studies, its closest homolog Flot1 (Yu et al., 2017) was reported to be present in similarly prepared plasma membrane fractions (Borner et al., 2005; Ishikawa et al., 2015). Additionally, rice Flot was also enriched when plasma membrane DRM fractions were prepared from rice (Ishikawa et al., 2015). Additionally, AHA1 and HIR2 together with AHA2, HIR1, and HIR4 were identified as major proteins tightly associated with a plasma membrane resistant to NaCl and Na_2_CO_3_ washing (Marmagne et al., 2007). Such a co-occurrence in specific plasma membrane sub-compartments may suggest a functional linkage in many cellular processes.

### Identified Interactors Suggest Putative Functions

*Flot2* transcription is highly upregulated upon bacterial, fungal, viral and oomycetal infection (Mohr and Cahill, 2007; Danek et al., 2016). Its transcription together with that of *SYP71*, *HIR2*, *NHL3* and *Flot3*, *HIR1*, *HIR3*, and *HIR4* were increased under viral infection (Ascencio-Ibanez et al., 2008). Increased *HIR2*, *Flot2* as well as *Flot1* transcription in mutants with altered systemic acquired resistance also suggests their involvement in this type of defense mechanism (Mosher et al., 2006). Moreover, the content of direct Flot2 interactors SYP71, AHA1, NHL3, ERD4, HIR2, and the HIR1 and HIR4 content in the plasma membrane DRM fraction was increased after treatment with the bacteria-derived elicitor flg22 (Keinath et al., 2010). Several of these interactors have been reported to be involved in resistance against pathogens.

Flot2 has already been found to directly interact with SYP71, a member of a plant-specific subfamily of Qc SNARE proteins (Sanderfoot et al., 2001). SYP71 transcription is increased under viral infection (Ascencio-Ibanez et al., 2008) and it plays a role in viral protein within the cell (Wei et al., 2013). Wheat and rice SYP71 homologs confer resistance to stripe rust and blast, respectively, and their transcription is upregulated upon infection by the respective pathogens, as well as upon treatment with hydrogen peroxide (Bao et al., 2012; Liu et al., 2016), a hallmark of hypersensitive plant defense (Coll et al., 2011). *Lotus japonicus* SYP71 is important for proper nodulation in *Mesorhizobium loti* symbiosis (Hakoyama et al., 2012). Interestingly, *M. truncatula* Flot2 and Flot4 are involved in nodulation, probably due to an interaction with an activated nodulation factor receptor (Haney and Long, 2010; Haney et al., 2011).

HIR2, another direct interactor of Flot2, belongs to the subfamily of SPFH proteins and is thus related to flotillins (Di et al., 2010). Four Arabidopsis HIRs interact with one another and HIR2 and HIR1 directly interact with the immune receptor RPS2. The interaction participates in effector-triggered resistance against *Pseudomonas syringae* (Qi et al., 2011). HIR homologs in pepper, rice and barley mediate the hypersensitive response to pathogens via an interaction with leucine-rich repeat proteins (Jung and Hwang, 2007; Zhou et al., 2009, 2010; Choi H.W. et al., 2011; Cheng et al., 2017).

NHL3 (NDR1/HIN1-LIKE 3) is another directly interacting protein involved in the plant–pathogen interaction. The transcription of NHL3 is induced by salicylic acid treatment, bacterial infection (Varet et al., 2002; Ditt et al., 2006), hydrogen peroxide treatment (Davletova et al., 2005) and by spermine, a polyamine signaling molecule inducing the expression of pathogenesis-related genes (Zheng et al., 2004). The overexpression of NHL3 leads to increased resistance to *P. syringae*. NHL3 was shown to be tightly associated with the plasma membrane (Varet et al., 2003), where it physically interacts with the oxidation-related zinc finger one protein that is also involved in salicylic acid-mediated defense reactions to bacterial attack (Singh et al., 2018).

The plasma membrane-localized H^+^ATPase AHA1 is a major proton pump contributing to stomata opening (Yamauchi et al., 2016), which makes it also closely connected with pathogen resistance reactions. AHA1 activity is altered by methyl jasmonate treatment (Yan et al., 2015) and binding to the bacterial effector AvrB (Zhou et al., 2015) or RIN4, a target of bacterial effectors (Liu J. et al., 2009). AHA1 together with HIR2 and HIR4 co-immunoprecipitated with HIR1 (Lv et al., 2017) and with Bax Inhibitor 1, an ER-localized suppressor of cell death after fungal infection (Weis et al., 2013). Interestingly, the overexpression of Bax Inhibitor 1 in rice cells resulted in the depletion of rice Flot and HIR homologs from DRM (Ishikawa et al., 2015). These findings suggest the involvment of HIRs, Flots and AHA1 in a shared pathway controling cell death and/or the reaction to pathogen attack.

We observed a direct interaction with two (PIP1-2 and PIP2-6) of five PIPs co-immunoprecipitating with Flot2. *PIP1-2*, *PIP2-1*, *PIP2-2*, and *PIP2-7* transcription is significantly decreased under drought stress, whereas *PIP2-6* transcription does not change (Alexandersson et al., 2005). PIP1-2 alone contributes, but due to functional redundancy with other PIPs, it is not essential for plant growth or water transport (Postaire et al., 2010); however, it is important for CO_2_ permeability and thus for the net photosynthesis rate (Heckwolf et al., 2011; Uehlein et al., 2012).

Besides this, the proper cellular trafficking of PIP2-7 and PIP2-2 between endomembranes and the plasma membrane is dependent on direct interaction with SYP121 and SYP61 (Hachez et al., 2014). A similar requirement was reported for maize PIP2-5 (Besserer et al., 2012). SYP121 also directly interacts with PIP2-2 (Besserer et al., 2012) and co-immunoprecipitates with SYP71 as well as some other SYPs, and, moreover, with HIR2 (Fujiwara et al., 2014). In addition, functional aquaporins are formed as tetramers, where the hetero-tetramerization of several single PIP isoforms has been reported (Jozefkowicz et al., 2017). The hetero-oligomerization of PIP1 and PIP2 group aquaporins is necessary for the trafficking of maize PIP1s to the plasma membrane (Zelazny et al., 2007).

PIP2-1 and PIP1-2 interact with several hundred proteins. Physical interaction was confirmed for PIP2-1 and NHL3 (Bellati et al., 2016), and three HIRs were detected to co-immunoprecipitate with at least one of PIP2-1 and PIP1-2. Therefore, a putative indirect linkage between PIP2-1, PIP2-2, PIP2-7, and Flot2 may be realized via PIP2-6 and PIP1-2, via SYP71 and SYP121 (with or without the involvement of HIR2), or via NHL3 or HIR2. Moreover, *PIP2-1* transcription in roots is decreased upon exposure to NaCl (Boursiac et al., 2005) and PIP2-1 was observed to be endocytosed from the plasma membrane upon NaCl treatment via Flot1-mediated endocytosis (Li et al., 2011). A similar involvement of Flot1 in clathrin-independent endocytosis was observed for the ammonium transporter AMT1-3 (Wang et al., 2015). Interestingly AMT1-3 was also found to interact with several Flot2 interactors determined in our study (Bellati et al., 2016). The function of Flot1 endocytosis remains unclear, but based on the very close similarity of both isoforms, it is possible that a Flot2-based complex can be implicated in similar processes.

AHA1 also contributes to water transport by its direct interaction with phytosulfokine receptors PSKR1 and PSKR2 and cyclic nucleotide-gated channel 17, a receptor for cyclic guanosine monophosphate. The application of both signal ligands leads to increased water influx, which is important for the volume growth of plant cells (Ladwig et al., 2015). Similarly, AHA1 is phosphorylated with the activated receptor of the peptide plant regulator PSY1R, which leads to root and hypocotyl elongation (Fuglsang et al., 2014). Proper AHA1 trafficking to the plasma membrane is important for plant growth, as plants with AHA1 accumulated in their endomembranes are smaller in size (Hashimoto-Sugimoto et al., 2013).

The participation of Flot2 in water management is also suggested by the direct interaction between Flot2 and ERD4, a member of the ERD protein family (Kiyosue et al., 1994). Enhanced tolerance to drought and salt stress was observed in Arabidopsis overexpressing maize *ERD4* (Liu Y. et al., 2009).

Although the identification of Flot2 specific interactors can suggest a potential role of Flot2 in *A. thaliana*, further studies are required. Phenotypic analysis of *Flot2* loss-of function mutants could be a valuable approach. We initially tested several abiotic and biotic treatments using a *flot2* T-DNA insertion line but did not observe any major effect different from wild type plants. Since there are three isoforms of flotillins encoded in *A. thaliana*, functional redundancy of these single isoforms may be an explanation for this lack of phenotype. Generation of multiple mutants may thus be necessary.

### Flot2 Interactome Forms a Complex Interlinked Network

**FIGURE 5:**
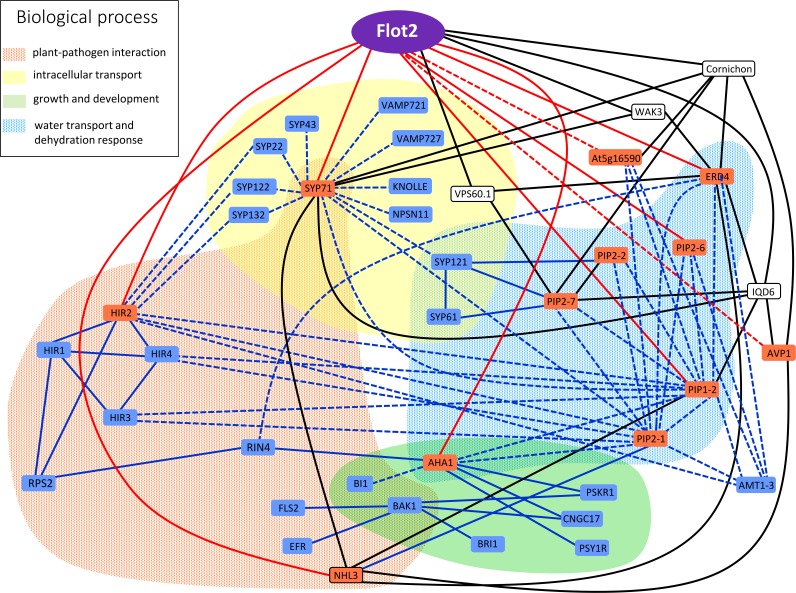
Graphical representation of Flot2 interaction network. Interactors experimentally determined in our study are displayed in red boxes. Solid red lines represent direct binding with Flot2 verified by SUS, while dashed red lines depict putative indirect interactions with Flot2 with negative SUS results. Blue boxes and lines represent direct (solid lines) or indirect (dashed lines) interactions published in literature and mentioned in discussion. Pair–wise interactions confirmed by yeast two hybrid or split ubiquitin assay, bimolecular fluorescence complementation or FRET/FLIM measurement are assumed as direct. Proteins found in co-IP assays and determined by mass spectrometry or revealed in tagged-protein pull-down assays, which were neither tested nor confirmed to be direct (by the methods listed above), were considered putative indirect interactors. Black boxes and lines represent most common direct interactions retrieved from Associomics database, which are common to several proteins detected in our study as well as Flot2. The implication of interactors in important biological processes is distinguished by colored fields.

As has already been pointed out, proteins found to interact with Flot2 in this study may also in many cases interact with each other, and thus it is possible that a given protein may be involved in several cellular functions. The situation gets even more complicated when direct interactors of each protein retrieved from the Associomic database are added to the list (**Figure 5**). Interestingly, it could be seen in Associomics that many of the Flot2 interactors found in this study also interact with other Flot2 interactors listed in Associomics. The most common interactors are NHL3, cornichon and IQD6. Since these proteins have several hundred interactors in Associomics, they could serve as real docking hubs for many proteins in the plasma membrane, and could thus be the crossroads or signposts of many pathways. On the other hand, it is possible that these proteins might be just too prone to giving false positive results. Intriguingly, the direct interactions of Flot2 that we confirmed in our study are not proposed for Flot2 in Associomics. Similarly, the direct interaction of NHL3 with PIP2-1 published in Bellati et al. (2016) is not found in Associomics. This comparison in fact demonstrates the general importance of the IP approach to membrane protein interactome determination.

## Author Contributions

JB, DK, and MD generated plant material. PJ, MH, RH, and OV contributed to the proteomic analysis and evaluation of results. MD, EZ, and KK prepared and performed SUS assays. PJ, MD, MJ, OV, and JM wrote the manuscript. PJ and MD contributed equally to this article.

## Conflict of Interest Statement

The authors declare that the research was conducted in the absence of any commercial or financial relationships that could be construed as a potential conflict of interest.

## Footnotes

1 https://associomics.dpb.carnegiescience.edu/Associomics/Home.html

2 http://www.uniprot.org

3 http://david.ncifcrf.gov
