# Supplementary material for: Mapping of Plasma Membrane Proteins Interacting With Arabidopsis thaliana Flotillin 2
Source: Front Plant Sci. 2018 Jul 12;9:991. doi: 10.3389/fpls.2018.00991 (PMC6052134; doi:10.3389/fpls.2018.00991)
Supplement: Supplementary file 1 [file Image_1.PDF]

Supplementary Material

Mapping of Plasma Membrane Proteins Interacting with *Arabidopsis thaliana* Flotillin 2

Petra Junková[1*†], Michal Daněk[2,3†], Daniela Kocourková[2], Jitka Brouzdová[2], Kristýna Kroumanová[2], Enric Zelazny[4], Martin Janda[1,2], Radovan Hynek[1], Jan Martinec[2], Olga Valentová[1]

[1]Department of Biochemistry and Microbiology, University of Chemistry and Technology Prague, Czech Republic

[2]Institute of Experimental Botany of the Czech Academy of Sciences, Prague, Czech Republic

[3]Department of Experimental Plant Biology, Faculty of Science, Charles University, Prague, Czech Republic

[4]Institute for Integrative Biology of the Cell (I2BC), CNRS/CEA/Université Paris Sud, Université Paris-Saclay, Gif-sur-Yvette, France

*** Correspondence:**
Petra Junková, PhD
Petra.Junkova @vscht.cz

† Contributed equally

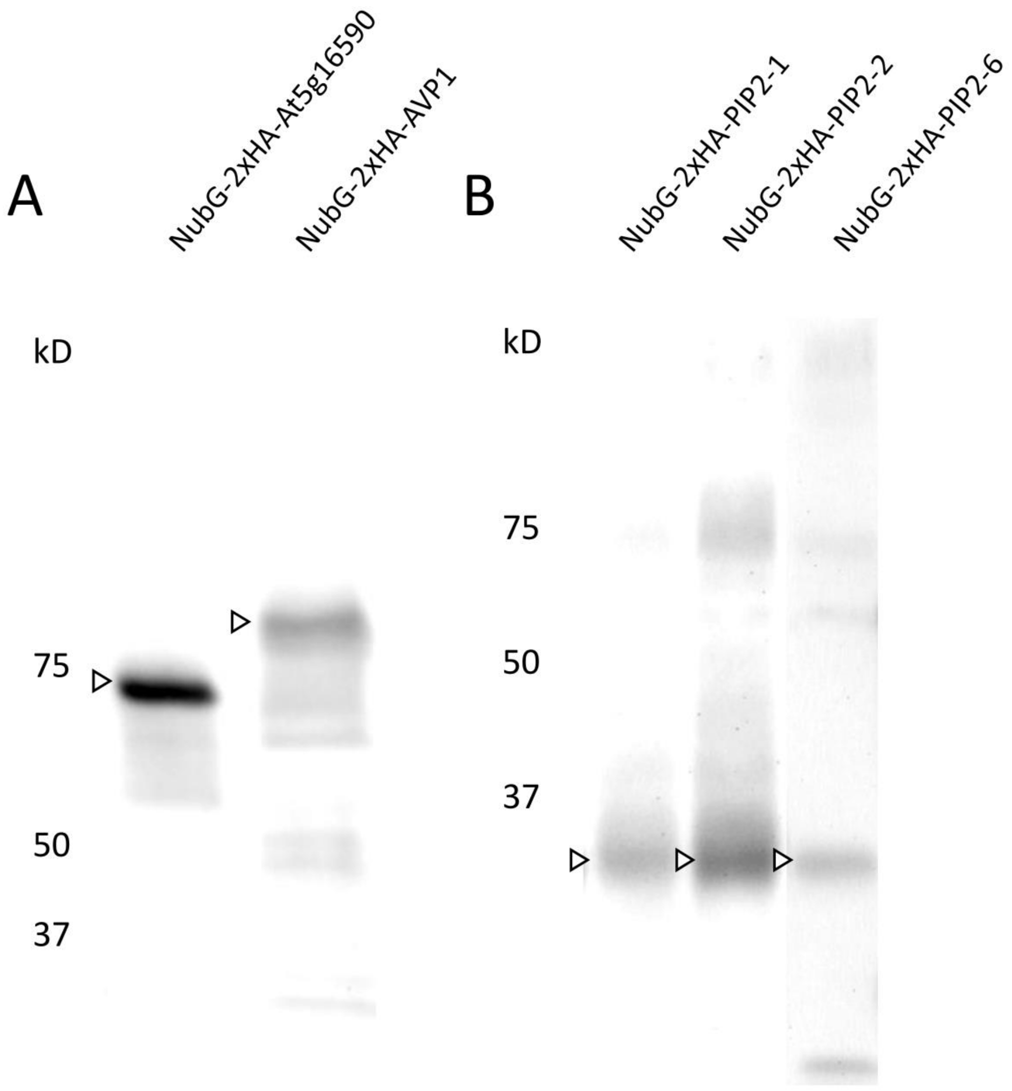

Supplementary Figure 1. Expression of fusion proteins in yeasts. Bands of corresponding molecular weight are marked with arrows. Proteins were separated on SDS-PAGE and blotted onto a nitrocellulose membrane using wet transfer. Membranes were probed with anti-HA primary antibody (Biolegend, cat. no. 901501) (1:2000, 1.5 h) and anti-mouse (1:5000, 1 h) secondary antibody. Panel A - Yeast cells were disrupted in lysis buffer (400 mM sucrose, 50 mM HEPES, 100 mM KCl, 100 mM $MgCl_2$, cOmplete Protease inhibitor Cocktail, 1mM DTT, pH 7.5) and centrifuged for 10 min at 6010 x g. The supernatant was further centrifuged for 1h at 27 460 x g in 4 °C. The pellet was resuspended in 1xPBS containing 5 % glycerol and used for immunoblotting. 15 µg of proteins were loaded into lanes and separated on 4-15 % SDS-PAGE. Panel B - Crude protein extracts were prepared according to (Grefen, 2014) and 10 µl of each sample was separated on 10 % SDS-PAGE.
